# Copolyamide-Imide Membrane with Low CTE and CME for Potential Space Optical Applications

**DOI:** 10.3390/polym13071001

**Published:** 2021-03-24

**Authors:** Jiajia Yin, Danbo Mao, Bin Fan

**Affiliations:** Institute of Optics and Electronics, Chinese Academy of Sciences, Chengdu 610209, China; yinjj@ioe.ac.cn (J.Y.); maodanbo@ioe.ac.cn (D.M.)

**Keywords:** copolyamide-imide membrane, optical homogeneity, thermal expansion behavior, moisture expansion behavior

## Abstract

Polyimide diffractive membrane lens can be used in space optical telescope to reduce the size and mass of an imaging system. However, traditional commercial aromatic polyimide membrane is hard to meet the challenging requirements of dimensional stability and optical homogeneity for optical use. Based on molecular structure design and the optimization of fabrication process, the prepared copolyamide-imide membrane achieved the desired performance of membrane as an optical material. It showed a very low coefficient of thermal expansion (CTE), which is 0.95 ppm/°C over a temperature range of −150–100 °C and relatively low coefficient of moisture expansion (CME), which is only 13.30 ppm/% RH (0~90% RH). For the optical use, the prepared copolyamide-imide membrane (φ200 mm) achieved good thickness uniformity with wave-front error smaller than λ/30 (λ = 632 nm) in RMS (root mean square). Besides, it simultaneously meets the optical, thermal, and mechanical requirements for space telescope use. Copolyamide-imide membranes in this research with good comprehensive performance can be used as large aperture membrane optical system architectures.

## 1. Introduction

Aromatic polyimides are a kind of high performance polymer with excellent mechanical properties, thermal stability, high UV/gamma radiation resistance, and chemical resistance [1,2,3]. It has been widely used in aerospace, solar cell, microelectronics, and display devices. It’s also an attractive large aperture lens candidate material due to their low surface density, solar radiation resistance, and cryogenic flexibility. It has been used to build space-based telescopes in several large optics systems [4,5,6,7,8,9]. For example, in MOIRE (Membrane Optical Imager Real-time Exploitation) program, patterned polyimide membrane has been used as transmissive diffractive membrane optic for space telescopes. In Falcon SAT-7 system, patterned polyimide membrane has been developed as a solar telescope. The diffractive polyimide membrane being used as light weighted primary optic has been considered as key elements enabling future earth observation and space science. In considering the transportation, it can not only significantly reduce the space vehicle lift capability but also reduce the program cost. In the aspect of optical system design, the optical path length error of diffraction membrane optics is virtually eliminated when light passes through the membrane, so the sensitivity to surface deformation is remarkably lowered compared with the reflective optical system, which is usually built by conventional quartz lens.

However, it is difficult for traditional commercial aromatic polyimide (PI) membrane to be used as a transmissive diffractive optical lens. They are hard to simultaneously meet the challenging requirements of high dimensional stability, good optical transmission, excellent mechanical property, and high temperature resistance. And among all the requirements, dimensional stability is the most important factor of materials for diffractive optical use. In the process of material synthesis, storage, structure manufacturing, ground prototype research, and development, PI membranes will face different thermal and moisture environment. Like under the ground-based researching stage, the humidity changes of the environment can tighten or slacken the membrane surface, changing its ability to handle loading [10]. While under the space thermal alternation application environment, the temperature change can lead to deformation of the microstructure on the membrane, then cause image distortion. A membrane optic is an optical system component with a large size, small thickness, and optical precision, so the membrane material and manufacturing process are nontrivial.

Therefore, it is urgent to develop an optical level PI membrane which applied as light-weight optics to have excellent dimensional stability. The coefficient of thermal expansion (CTE) and coefficient of moisture expansion (CME) were key capability indexes representing the dimensional stability of PIs. Significant effort has been spent in synthesizing low CTE/CME PIs that still maintain their excellent mechanical, optical, and thermal properties by structural modification. In this research, to meet with the high requirements for dimensional stability, the CTE of the polyimide membrane was adjusted near zero and the CME of polyimide membrane has been lower down to 13.3 ppm/RH%. At the meantime, the excellent optical, mechanical, and thermal properties of polyimide membrane have been maintained. More importantly, by optimizing the preparation process, the optical homogeneity has been guaranteed. Compared with traditional aromatic polyimide, the designed copolyamide-imide (co-PAI) is a good candidate for potential space optical applications.

## 2. Materials and Methods

### 2.1. Materials

4,4′-Diaminobenzanilide (DABA, 98%), 4,4′-Diamino-2,2′-Dimethylbiphenyl (TMDB, 98%), 3,3′,4,4′-Biphenyltetracarboxylic Dianhydride (BPDA, 98%), Pyromellitic Dianhydride (PMDA, 98%), and anhydrous *N*-Methyl-2-Pyrrolidinone (NMP, 99%) were purchased from TCI reagents (Shanghai, China). PMDA and BPDA were dried at 180 °C in a vacuum (Reale, Dongguan, China) for 24 h prior to use. NMP was purified by distillation under reduced pressure and dehydrated with 4Å molecular sieves prior to use. Other solvents and regents were used as received.

### 2.2. Preparation of Copolyamide-Imide Membrane

**Figure 1:**
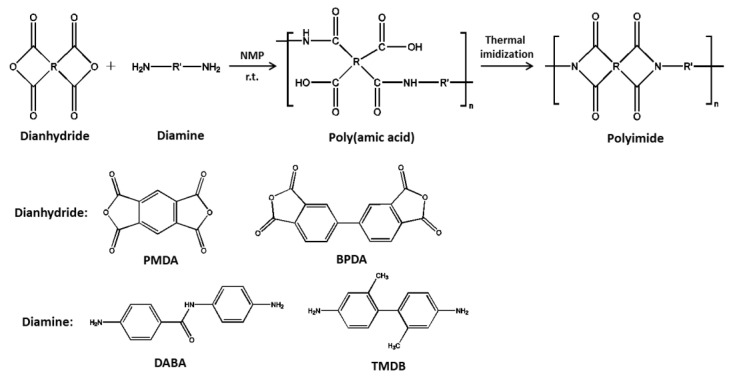
The synthesis of Poly(amic acid) and preparation process of copolyamide-imide (co-PAI) membrane.

Poly(amic acid) (PAA) resin was synthesized by the polyaddition of equimolar amounts of diamine (DABA/TMDB; molar ratio DABA:TMDB = 1:1) and dianhydride (BPDA/PMDA; molar ratio BPDA:PMDA = 1:1). Then, co-PAI membrane was prepared followed by thermal imidization, as shown in Figure 1. In the experiment, co-PAI membrane was prepared according to the following procedure. Diamine DABA/TMDB were dissolved in NMP in a dry three neck flask equipped with a mechanical stirrer (Taihongsheng, Zhengzhou, China) and nitrogen flow under room temperature. Then, dianhydride BPDA/PMDA were added to the solution in batches with continuous stirring. The reaction mixture was stirred for 30 min at 0 °C and then left to react overnight at room temperature. The concentration of the solution will be controlled around ~10% (wt). The homogeneous and viscous PAA resin was produced with a high inherent viscosity of 1.34–1.75 dL/g, which indicates that the polymeric precursor have a relatively high molecular weight [11,12,13].

The PAA resin was filtrated and deaerated before casted on the surface of a quartz glass plate by spin coating (Leibo, Jiangsu, China). The wet membrane will be pre-imidized by a hot plate under 70 °C for 30 min to remove excess solvent. Then, it was heated by a vacuum oven (Reale, Dongguan, China) in stages to elevated temperatures to further remove solvent and convert the amic acid functional groups to imides with a cyclodehydration reaction, the detailed thermal imidization procedure is shown in Figure S1 (Supplementary Materials, Figure S1). The rising and cooling process has been optimized according to the previous study [14,15,16] to ensure the fully imidization of co-PAI. In the current work, to ensure the optical uniformity of a large diameter co-PAI membrane for optical use, spin coating and thermal imidization procedure will be repeated three times. The thickness of the membrane will be around 22 μm. The produced polyimide membrane was separated from the substrate by customized equipment and annealed at an established temperature to release the residual stress.

### 2.3. Characterization

The optical inhomogeneity of membranes was characterized by a wave-front error using a Zygo laser interferometer (GPI XP, Middlefield, CT, USA) with an accuracy of λ/1000 (λ = 632.8 nm, λ is the measurement wavelength). The thickness of the polyimide films was measured by using a commercially available spectral reflectometer (Filmetrics-F20 thin film analyzer, Filmetrics Inc., San Diego, CA, USA). The surface roughness was measured by laser interferometer optical microscope (Bruker Optics, Ettlingen, Germany). The transmission spectra of membranes (22 μm thickness) was measured by an ultraviolet-visible-near infrared spectrophotometer (Lambda 1050, Perkin Elmer, Waltham, MA, USA) in the wavelength (λ) range of 200–800 nm. The in-plane (n_TE_) and out-of-plane (n_TM_) refractive indices of membranes were measured on a Metricon 2010 prism coupler (Metricon Corporation, Pennington, NJ, USA) at the wavelength of 633 nm. The average refractive index (n_av_) and birefringence (Δn) values were calculated according to the following equation: n_av_ = [(2n_TE_^2^ + n_TM_^2^)/3]^1/2^. The partial structure profilometer was tested by Zygo New view 7300 white-light interferometer (Middlefield, CT, USA). The in-plane coefficients of thermal expansion (CTE) of membranes were carried out on a TA 450 EM thermo mechanical analysis (TMA) instrument (TA Instruments, New Castle, DE, USA) in a nitrogen atmosphere at a heating rate of 5 °C/min with a fixed load (0.05 N for 22 μm). The CTE values of samples were determined by calculating the results in a temperature range of −150–100 °C. CME values were obtained from the TA 450EM with humidity accessory. The CME values of samples were determined by calculating the results in a humidity range of 0–90% RH under room temperature. The glass transition temperature (T_g_) of PI specimens were regarded as the peak temperature of the tan δ curves, which was measured by the dynamic mechanical analysis (DMA) performed on a TA Q800 instrument (TA Instruments, New Castle, DE, USA) at a heating rate of 5 °C/min in nitrogen with a load frequency of 1 Hz in membrane tension geometry. The thermal stability of PI membranes was evaluated by thermo gravimetric analysis (TGA), which were performed on a TG 209 F_1_ Libra (Netzsch, Selb, Germany) at a heating rate of 20 K/min in a nitrogen atmosphere (40 mL/min). The values of onset, 10%, 20% weight loss temperatures (T_10_ and T_20_), and residue at 750 °C (R_w_) were obtained from the TGA curves. The tensile properties of PI membranes were measured on an Instron-5944 tensile apparatus (Norwood, MA, USA) with 150(l) × 20(w) × 0.022(h) mm specimens at room temperature in accordance with the Chinese national standard of GB/T1040.3-2006 at a drawing rate of 10 mm/min.

## 3. Results and Discussion

### 3.1. Optical Properties

**Figure 2:**
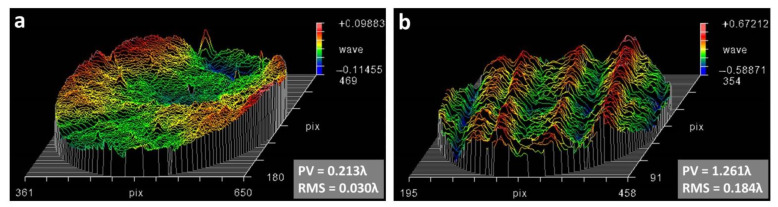
The wave-front error of (**a**) co-PAI and (**b**) commercial Kapton membranes.

Thickness uniformity and surface roughness [17] of transmissive diffractive membrane have a strong impact on optical homogeneity. They can influence the image quality of the diffraction optical system. But most of the commercial PI membranes can’t fulfill the optical homogeneity requirements. In general, for large batch production, commercial PI membrane has usually been fabricated by flow spreading or knife coating method. It is hard to get PI membrane with high precision thickness uniformity for optical use. In this research, multiple spin coating method has been applied to get optical quality PI membrane. Based on the previous work of our group [18], the resin application method, solid concentration, resin viscosity, spin speed, spin time, and procure temperature have been controlled within appropriate tolerances to achieve a desirable thickness membrane with good uniformity. Kapton series is a high performance PI membrane which has been widely used in the market. The thickness aberration of co-PAI and commercial Kapton PI membranes with 22 μm thickness and φ200 mm aperture have been characterized by wave-front errors with the application of the Zygo laser interferometer. As shown in Figure 2, the co-PAI membrane achieved good thickness uniformity with wave-front error smaller than λ/30 in RMS (root mean square), while commercial Kapton PI membrane acquired wave-front error of about λ/6 in RMS.

**Figure 3:**
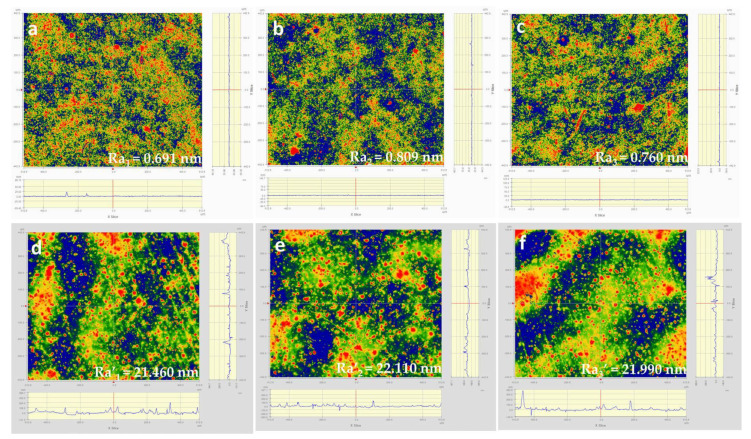
The surface roughness results of co-PAI (**a**–**c**) and commercial Kapton (**d**–**f**) in three different areas on each membrane.

A membrane with low surface roughness will avoid diffuse reflection of light. This parameter will directly influence the diffractive patterns fabrication precision. As shown in Figure 3, co-PAI has a surface roughness (Ra) under 1 nm, while commercial Kapton PI membrane showed a much rougher surface with a surface roughness (Ra’) above 20 nm.

**Figure 4:**
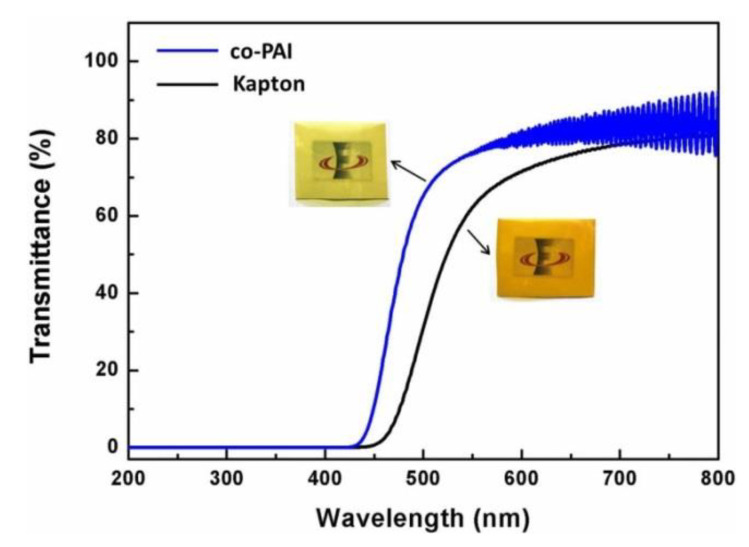
The UV–Vis transmittance spectra of co-PAI and commercial Kapton membrane. The insert pictures are the picture of two membranes, respectively.

Transmittance of optical lens decide the luminous flux, the SNR (signal to noise ratio), and stray light of optical system [11]. Traditional aromatic polyimide has a relatively low transmittance in the visible light range because of the formation of intra- and inter-molecular charge-transfer complex (CTC) in molecular structures [19,20]. Transmittance spectra of co-PAI and commercial Kapton membrane were plotted in Figure 4. As shown in the picture, co-PAI showed a better transmission than Kapton in the visible range, especially in the window of 500–800 nm, which is important to space telescope for ground observation. The average transmittance of co-PAI in this window is 80%, while for the Kapton membrane it is only 69%. The cut off wavelength (λ_0_) of co-PAI membranes is 434 nm. In contrast, the Kapton membrane with deeper yellow appearance performed higher λ_0_ of 454 nm. The data has been summarized in Table 1.

The refractive index is an important parameter for transmissive diffractive optics. It’s necessary for Fresnel diffractive structure fabrication. For instance, to fabricate the microstructure on membranes, the optimal total etched depth, d, for an m-level profile is d = (m − 1)λ/[m(n − 1)], where n is the refractive index of the material and λ the design wavelength [21]. The refractive performance of co-PAI and Kapton membranes were measured by the prism coupling method, and refractive parameters including refractive indices (n_TE_ and n_TM_), average refractive indices (n_av_), and birefringence (∆n) values were summarized in Table 2. The average value of co-PAI membrane is 1.7626, which is at the similar level compared with typical aromatic polyimide membrane, such as the Kapton membrane (1.7110). The similar refractive index makes it possible to refer to the existing lithography to fabricate Fresnel diffractive structure on flexible co-PAI membrane.

### 3.2. Dimensional Stability

**Figure 5:**
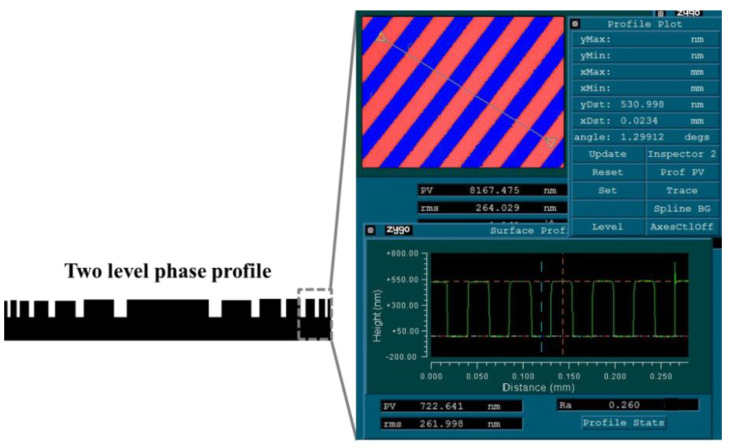
Schematic diagrams of a two level phase profile and partial structure profilometer test result.

Transmissive diffractive optic (two steps phase) is patterned on one surface with microstructures, as shown in schematic diagram (Figure 5, left). The microstructures are designed to bend light of particular wavelengths. The height of surface microstructure is on the order of the light wavelength. For example, in one of our designed membrane transmissive diffractive optic (Figure 5, right), the height of the surface microstructure is only 530 nm. It is known that the common aromatic PI membranes usually have a coefficient of thermal expansion values in the membrane plane direction of 10–60 ppm/°C and coefficient of moisture expansion values in the membrane plane direction of 20–30 ppm/RH%. The dimensional expansion of polyimide membrane substrate will result in the shape change of microstructure on the membrane, like the size change of etching depth, etching width, and structure position on large aperture Fresnel zone lens. It will result in image distortion [22,23,24]. Additionally, the temperature of large aperture membranes is not uniformly controlled in space, further increasing image distortion from anisotropic deformations. So, the PI membrane with improved dimensional stability is highly appreciated.

**Figure 6:**
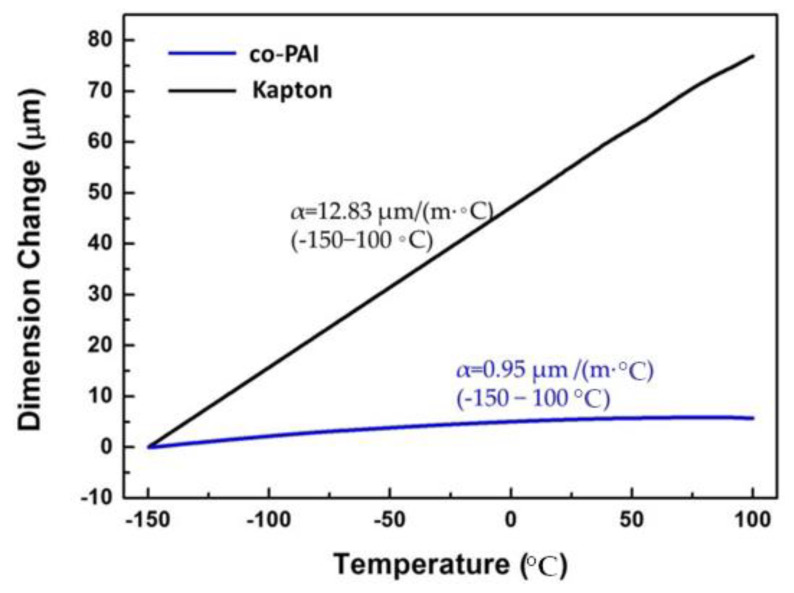
Thermal dimensional stabilities of the co-PAI and commercial Kapton membrane (temperature range: −150–100 °C).

The high CTE of polyimides usually arises from the arrangement of carbon-carbon bonds in polymer skeleton. The arrangement of carbon-carbon bonds determines the capacity for converting absorbed thermal energy to crankshaft rotation. Crankshaft rotation sweeps out free volume and produces bulk physical expansion with increased temperature [25]. The thermal expansion behavior of PI membranes was investigated by TMA. The dimensional changes over a broad temperature range of −150–100 °C were shown in Figure 6. The CTE value of co-PAI is on the order of quartz (0.5 ppm/°C). And it is much lower than that of commercial Kapton membrane (12.83 ppm/°C), which is only 0.95 ppm/°C. The low CTE value of co-PAI owns to the hydrogen bonding formation between the proton-donors (N–H) and electron-rich groups (C=O or C=N) [26,27,28]. Hasegawa and other researchers also find these similar results from the PIs containing ester or amide groups in the backbone structures.

**Figure 7:**
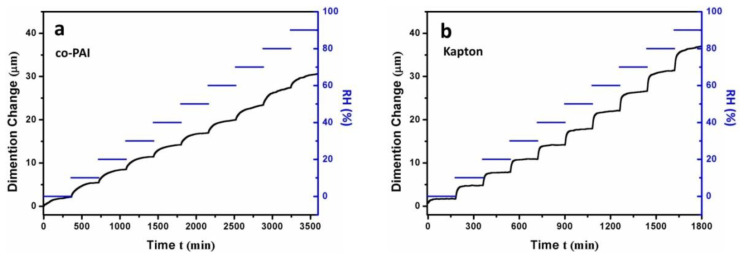
Humidity dimensional stabilities of the (**a**) co-PAI and (**b**) commercial Kapton membranes under room temperature (25 °C).

**Figure 8:**
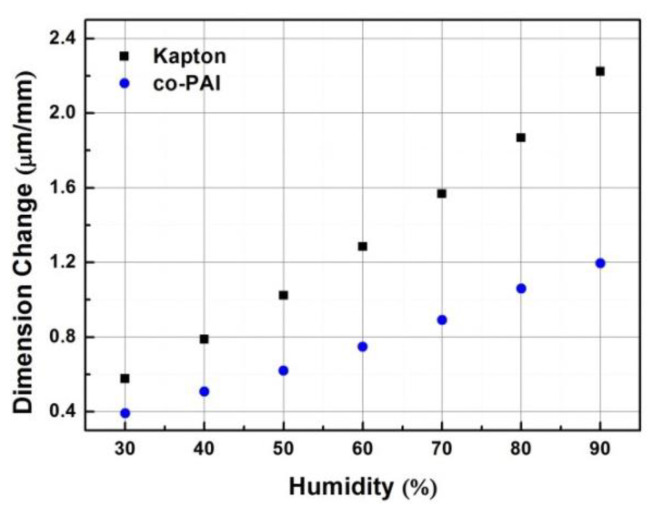
The conclusion of humidity dimensional changes of the co-PAI and commercial Kapton membranes on each level under room temperature (25 °C).

The high CME of polyimide arising from the hydrophilic imide rings, nanoporous inner structure, and some hydrophilic moieties (ether, sulfone, and carbonyl group, etc.) in the polymer backbones [29]. The humidity expansion behavior of PI membranes was investigated by TMA with humidity accessary over a range of 0% RH–90% RH under room temperature (25 °C). Different from heat conduction, the wet conduction behavior usually takes a longer time to reach the balance, so the samples were kept in each humidity level for 3 h or more. The results are shown in Figure 7. Compared with commercial Kapton membrane (24.6 ppm/% RH), co-PAI showed a much lower CME, which is 13.3 ppm/% RH over a humidity range of 0% RH–90% RH. As shown in Figure 8, at each humidity level (10% RH; 20% RH; 30% RH; 40% RH; 50% RH; 60% RH; 70% RH; 80% RH; 90% RH), the co-PAI membrane showed much better moisture dimensional stability than Kapton membrane as shown in Figure 8.

### 3.3. Thermal and Mechanical Properties

**Figure 9:**
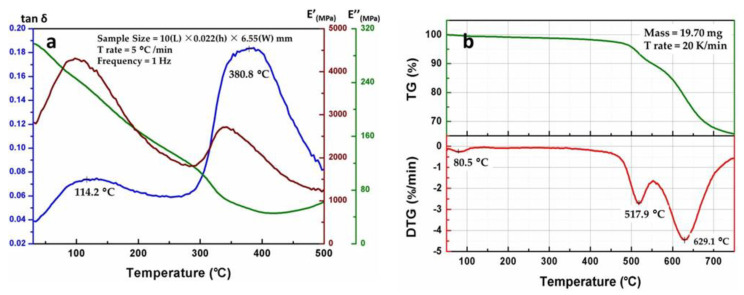
(**a**) Dynamic mechanical analysis (DMA) curve and (**b**) thermo gravimetric analysis (TGA) curves of the co-PAI membrane.

The alternative thermal environment is also a challenge for PI membrane thermal stability. The glass transition temperature (T_g_) of co-PAI is regarded as the peak temperature of the tan δ curve in DMA. As shown in Figure 9a, co-PAI membrane exhibited excellent thermal stability with T_g_ values as high as 380.8 °C. The decomposition temperatures of it at T_10%_ and T_20%_ in nitrogen are 540 °C and 620 °C, respectively. The carbon yield at 750 °C is 65% (Figure 9b). The good thermal stability can be attributed to the intermolecular interaction between amide structures. The strong interaction restricted the rotation of segment movement, which helped to improve the heat resistance of the material.

**Figure 10:**
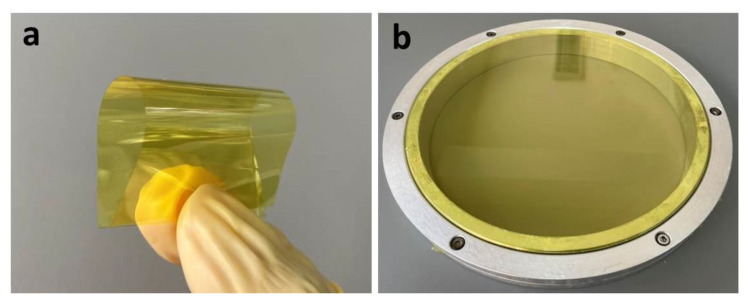
(**a**) Photo of co-PAI membrane (thickness = 22 μm); (**b**) membrane optic structure design.

**Figure 11:**
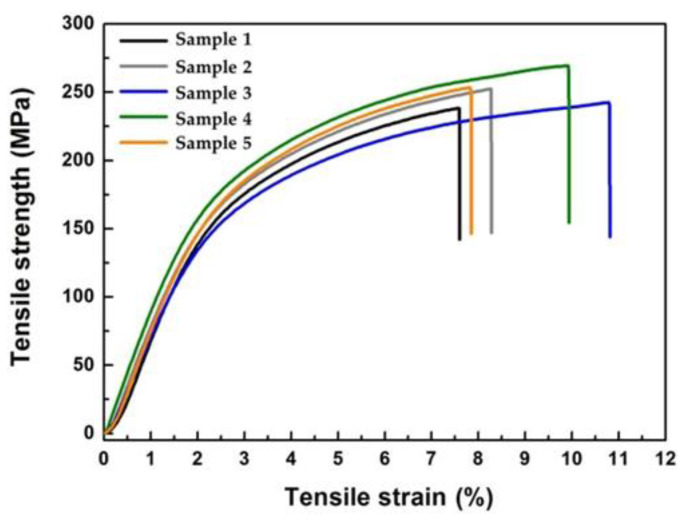
Tensile strength of co-PAI membrane (five samples).

Flexible co-PAI membrane (Figure 10a) needs to be mounted on the semi-rigid frame in the telescope optical design, as shown in Figure 10b. Good mechanical properties will avoid the wrinkles in the membrane due to the effects of the membrane tension. As shown in Figure 11, co-PAI presented excellent mechanical properties with average tensile strength of 251 MPa, Young’s modulus of 8.7 GPa, and elongation at break of 8.9%, respectively. The strong and tough performance of co-PAI could be owing to their rigid backbones combined with strong inter-molecular interactions.

## 4. Conclusions

Optical grade co-PAI membrane with excellent dimensional stability was successfully prepared. The co-PAI membrane exhibited an ultralow in-plane CTE 0.95 ppm/°C over a temperature range of −150–100°C. And it has a relatively low CME of 13.5 ppm/RH% over a humidity range of 0–90% RH (25 °C). The ultralow CTE behavior of co-PAI membranes was mainly caused by the highly oriented of linear/rigid main chains in the membrane plane and strong hydrogen bonding interactions. It also processed good heat resistance and thermal stability with T_g_ 380.5 °C and 10%/20% decomposition temperatures of 540 °C and 620 °C. At the same time, the co-PAI membrane showed good mechanical (251 MPa) and optical properties (T_500–800 nm_ = 80%). In this research, the multiple spin coating method has been used to get PI membrane with excellent optical homogeneity (PV = 0.213λ; RMS = 0.030λ) and good surface roughness (Ra under 1 nm). Compared with the traditional PI membrane, co-PAI is a good candidate for potential light-weight space optical imaging lenses. The preparation of optical grade polyimide membranes with good space environment performance and a diameter more than 400 mm is under way by our team and will be reported in the near future.

## Figures and Tables

**Table 1:** Optical transparency of co-PAI and commercial Kapton membranes.

Sample	λ_0_^1^ (nm)	T_500_^2^ (%)	T_800_^3^ (%)	T_av_^4^ (%)
co-PAI	434	64	82	80
Kapton	454	29	79	69

λ_0_^1^: UV cutoff wavelength; T_500_^2^, T_800_^3^: transmittance at 500 nm, 800 nm, respectively; T_av_^4^: average transmittance between 500 to 800 nm.

**Table 2:** Refractive indices of co-PAI and Kapton membranes.

Sample	n_TE_^1^	n_TM_^2^	n_av_^3^
co-PAI	1.8342	1.6099	1.7626
Kapton	1.7562	1.6169	1.7110

n_TE_^1^, n_TM_^2^: determined at 632.8 nm; n_av_^3^= [(2n_TE_^2^ + n_TM_^2^)/3]^1/2^.

## Data Availability

The data presented in this study are available on request from the corresponding author.

## Supplementary Materials

The following are available online at https://www.mdpi.com/2073-4360/13/7/1001/s1, Figure S1: Thermal imidization procedure.
