# Supplementary material for: Copolyamide-Imide Membrane with Low CTE and CME for Potential Space Optical Applications
Source: Polymers (Basel). 2021 Mar 24;13(7):1001. doi: 10.3390/polym13071001 (PMC8037737; doi:10.3390/polym13071001)
Supplement: Supplementary file 1 [file polymers-13-01001-s001.pdf]

Supplementary Materials

There are two stages for the thermal imidization procedure.

a. First is the stepped heating stage, the detailed heating rate showed as follow:

$$25℃ \xrightarrow[30\ min]{2.5℃/min} 100℃(1\ h) \xrightarrow[30\ min]{3.3℃/min} 200℃(1\ h) \xrightarrow[45\ min]{3.3℃/min} 350℃(1\ h)$$

b. Then is the cooling stage, the detailed cooling rate showed as follow:

$$350℃ \xrightarrow[420\ min]{-0.77℃/min} 25℃$$

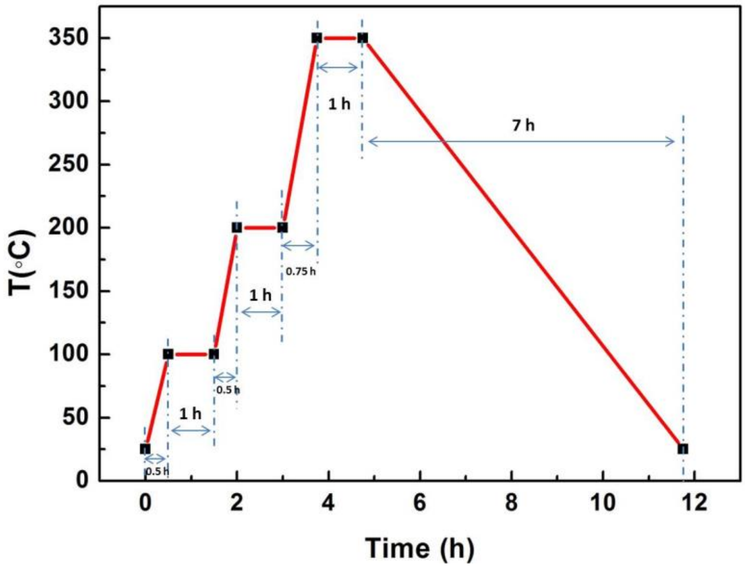

Figure S1. Thermal imidization procedure.
